# Precipitating Risk Factors, Clinical Presentation, and Outcome of Diabetic Ketoacidosis in Patients with Type 1 Diabetes

**DOI:** 10.7759/cureus.4789

**Published:** 2019-05-31

**Authors:** Warsha Ahuja, Navin Kumar, Sumeet Kumar, Amber Rizwan

**Affiliations:** 1 Internal Medicine, Jinnah Sindh Medical University, Karachi, PAK; 2 Internal Medicine, Chandka Medical College Hospital Larkana, Larkana, PAK; 3 Family Medicine, Dr. Ruth Pfau Hospital, Karachi, PAK

**Keywords:** diabetic ketoacidosis, diabetic ketoacidosis complications, mortality risk, type 1 diabetes, predictors of mortality, clinical spectrum, risk factors

## Abstract

Introduction

Over the past few years, there have been drastic advancements in the management of type 1 and 2 diabetes mellitus (DM). Prevention of complications is a prime concern of all physicians dealing with DM. However, whether or not these interventions have helped in reducing the incidence of diabetic ketoacidosis (DKA) in patients with type 1 DM, is still an unanswered question. The aim of this study is to assess the clinical pattern of DKA, evaluate its outcomes, and study the predictors of outcome.

Methods

The study was conducted as a prospective, observational one in the department of medicine of a tertiary care hospital from July-December 2018. Patients of type 1 DM presenting in the emergency department with DKA were evaluated for their predisposing factors, clinical presentation, biochemical parameters, rate of mortality, and predictors of mortality. Data was processed through and analyzed using IBM SPSS Statistics for Windows, version 22.0 (IBM Corp., Armonk, NY).

Results

The common clinical presentations include severe vomiting (32.2%), abdominal pain (27.9%), and depressed mental state (DMS) (26.8%). Infections (36.5%) and inadequate insulin dose (22.5%) were frequently seen as the predisposing factors. In one-fourth of the patients, this episode of DKA was the first presentation of DM (26.8%). The rate of mortality was 23.6%. The predictors of mortality included DMS, markedly low pH and serum bicarbonate, and high serum potassium at the time of presentation, random blood sugar >300 mg/dL and urine positive for ketones even after 12 hours of medical intervention, >50 international units (IU) insulin requirement within the first 12 hours, >6L fluid replenished within the first 24 hours, and new onset of fever within the first 24 hours.

Conclusion

The clinical presentation of DKA is not stark and vague signs such as generalized fatigue, nausea vomiting, abdominal pain, and DMS should raise suspicion. Underlying infections and inadequate insulin regimen predispose to acute DKA attack. Rate of mortality is high in these patients. Morality may be predicted by various clinical and biochemical parameters.

## Introduction

Diabetes mellitus (DM) is a complex endocrinological disorder with altered metabolism of blood glucose. It is a long-standing disease with both short term and long term complications. The most intense acute complication of diabetes include hyperosmolar hyperglycemic state and diabetic ketoacidosis (DKA). Both conditions require urgent medical interventions [1]. The mortality ratio with hyperglycemic emergencies has been variable depending on the geographical location. In developing countries, the incidence of mortality may range from 4%-40% [2-5].

DKA renders the body to severely altered metabolism of not only carbohydrates but also proteins and fats. The body goes into a mode of catabolism with rapid breakdown of glycogen stores, triglyceride hydrolysis and amino acid mobilization resulting in subsequent production of glucose and ketone bodies by the liver which further aggravates the metabolic decompensation [6].

The two most frequently encountered risk factors precipitating to DKA are missed insulin dose and presence of infection [4]. Although, any factor causing stress on the body, such as myocardial infarction, stroke, trauma, and substance abuse, may result in DKA [7]. Another striking cause of DKA is the first presentation of diabetes. As many as 33% of undiagnosed DM cases present with DKA at the first instance [8]. DKA may ensue within a few hours of the precipitating factors. However, since most of its initial signs are non-specific; diagnosis may be delayed. The clinical features of DKA include, but are not restricted to, nausea, severe vomiting, dehydration, abdominal pain, acetone breath, Kussmaul breathing pattern, and generalized fatigue. Despite underlying infection, some patients maintain normal body temperature or even hypothermia in DKA [6]. Clinical outcome in DKA is largely dependent on the patient’s response to initial medical interventions within the emergency room and within the first 24 hours after admission, precipitating illness, and the biochemical profile [4]. Depressed mental state (DMS)/comatose state at the time of presentation and/or within the first 24 hours has also been identified as an independent predictor of mortality in DKA [9].

Despite a gradual rise in the incidence of diabetes in Pakistan, both type 1 diabetes and its complications are not readily discussed in the literature. Very limited literature is available regarding DKA in Pakistani individuals with type 1 diabetes; most of it deals with pediatric population [10-11]. For enhancing the existing management strategies entailing DKA in young Pakistani diabetic population, it is crucial for the medical specialists to evaluate the spectrum of clinical characteristics of DKA, its outcomes, and also the factors influencing those outcomes. This study aims to assess the trend of clinical features of DKA, evaluate the outcomes, and study the predictors of outcome in type 1 DM patients with DKA.

## Materials and methods

The study design was prospective, observational. It was conducted in the department of medicine of the largest tertiary care hospital in Karachi, Pakistan. The study duration was July-December 2018. The study was approved by the Institutional Review Board which exempted patient consent.

**Figure 1:**
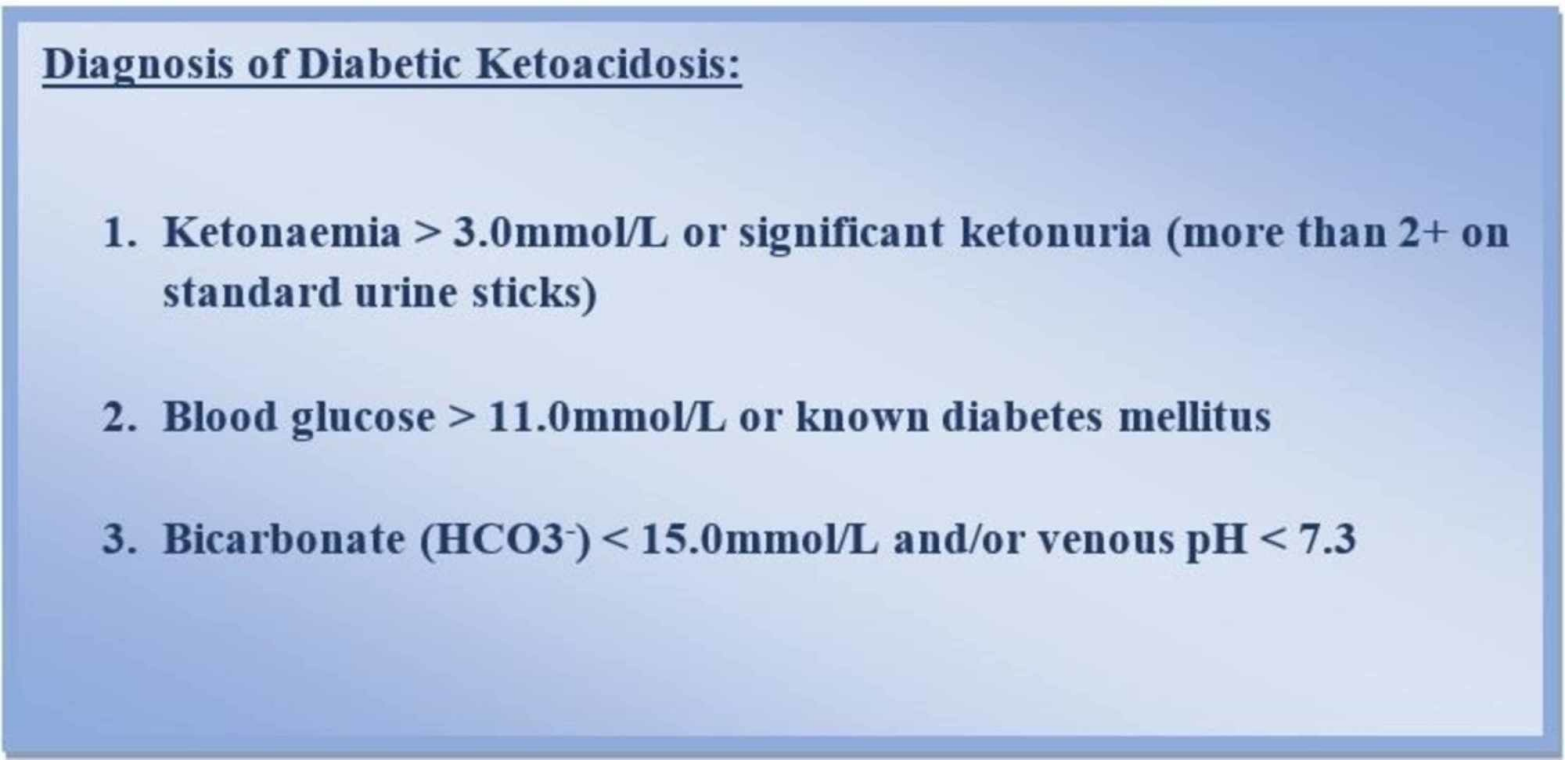
Diagnostic Criteria of Diabetic Ketoacidosis

The inclusion criteria comprised of patients of type I diabetes or undiagnosed cases (first presentation) presenting with DKA in the emergency department. DKA was defined as a biochemical trial of ketonemia, academia, and hyperglycemia as shown in Figure 1.

The diagnosis, as well as management of DKA in our study, was according to the International Society for Pediatric and Adolescent Diabetes (ISPAD) Clinical Practice Consensus Guidelines, 2018 [12].

All patients were followed throughout their hospital stay to evaluate their clinical spectrum. Clinical presentation and precipitating risk factors were noted for all patients. Each patient may have presented with more than one symptom; the most prominent one was taken for each patient. Clinical and biochemical parameters including serum potassium (mEq/L) and bicarbonate (mmol/L) and blood pH at the time of presentation, insulin requirement during the first 12 hours (International Units; IU), random blood sugar (RBS) after first 12 hours (mg/dL), total fluid requirement during the first 24 hours (Liters), development of fever within first 24 hours with no fever at presentation, and urinary ketones after the first 24 hours were recorded for all patients and compared with patient outcome - recovery or mortality. Only patients who survived for at least the first 24 hours were included in the analysis. Acute complications including acute renal failure, acute respiratory distress syndrome (ARDS), and cerebral edema were recorded for all patients. Causes of deaths were recorded for patients in the mortality group.

Data was processed through and analyzed using IBM SPSS Statistics for Windows, version 22.0 (IBM Corp., Armonk, NY). Mean and standard deviation (SD) were calculated for continuous variables. Frequency and percentages were calculated for categorical variables. Comparison was done for clinical presentation and precipitating risk factors with age and gender. Mortality predictors were compared for recovery and mortality group. Odds ratio and confidence interval (CI) was calculated. P value of ≤0.05 was taken as significant.

## Results

Ninety-three participants were included in the study. Their mean age was 19 ± 7 years (range: 7-24 years). Forty-five (48.4%) patients were of age less than 18 years and the remaining 48 (51.6%) were above the age of 18 years. There were 39 (41.9%) women and 54 (58.1%) men. There were 25 (26.8%) patients with first presentation of diabetes. Other than that, the mean duration of diabetes was 72 ± 13 months (range: 5-120 months).

The clinical presentation of DKA was diverse and one patient had one or more symptoms. The most prominent symptom for each patient was taken. As seen in Table 1, 32.2% presented with severe vomiting, 27.9% with abdominal pain, 26.8% with DMS/comatose condition, 8.6% with fever, and 4.3% presented with polyuria/polydipsia. Severe vomiting was more common in the older age group and in women. Abdominal pain was more common in the younger group of patients and in men. DMS/comatose condition was more commonly encountered in women. The differences were statistically significant as shown in Table 1.

**Table 1:** Patient Symptoms, According to Age and Gender, at the Time of Presentation of Diabetic Ketoacidosis (N=93) DMS, Depressed mental state.

Presenting symptoms	Age <18 years	Age >18 years	P value	Men	Women	P value	Total patients
Severe vomiting	10 (33.3%)	20 (66.7%)	0.04	13 (43.3%)	17 (56.7%)	0.04	30 (32.2%)
Abdominal pain	18 (69.2%)	8 (30.8%)	0.02	20 (76.9%)	6 (23.1%)	0.02	26 (27.9%)
DMS / comatose	13 (52.0%)	12 (48.0%)	0.67	9 (36.0%)	16 (64.0%)	0.008	25 (26.8%)
Fever	2 (25.0%)	6 (75.0%)	0.16	8 (100.0%)	0	---	8 (8.6%)
Polyuria / polydipsia	3 (75.0%)	1 (25.0%)	0.27	4 (100.0%)	0	---	4 (4.3%)

All patients were assessed for the risk factors which predisposed to the development of DKA. We observed that in 36.5% patients, there was an underlying infection. In 26.8% patients, this emergency was the first presentation of diabetes and 22.5% patients had taken inadequate dose/missed their insulin dose. In 13.9% cases, no risk factors could be identified as seen in Table 2. We also observed that underlying infections were more likely to be in the older age group. First presentation of diabetes was associated with younger age group. The results were statistically significant (Table 2).

**Table 2:** Risk Factors Precipitating to Diabetic Ketoacidosis (N=93)

Precipitating risk factors	Age <18 years	Age >18 years	P value	Men	Women	P value	Total patients
Underlying infection	7 (20.5%)	27 (79.4%)	0.000	20 (58.8%)	14 (41.2%)	0.91	34 (36.5%)
First presentation of diabetes	22 (88.0%)	3 (12.0%)	0.000	17 (68.0%)	8 (32.0%)	0.23	25 (26.8%)
Inadequate dose of insulin	13 (61.9%)	8 (38.1%)	0.15	12 (57.1%)	9 (42.8%)	0.92	21 (22.5%)
Other/unknown	3 (23.0%)	10 (77.0%)	0.04	5 (38.4%)	8 (61.5%)	0.12	13 (13.9%)

All seven individuals of age less than 18 years with underlying infections had urinary tract infection (UTI). In the older age group, nine (33.3%) had a lower respiratory tract infection (RTI), seven (25.9%) had a UTI, six (22.2%) had an infected toe nail, three (11.1%) had gastroenteritis, and two (7.4%) had cellulitis from repeated intravenous (IV) cannulation for IV glucose replacement in settings of severe hypoglycemia.

We then evaluated the in-hospital outcome of these patients with DKA. The mortality rate in our study was 22 (23.6%) and the remaining 71 (76.3%) recovered well. Among the patients who died, 13 (59.0%) developed septic shock, six (%27.3) died of hypokalemic arrhythmia, and three (13.6%) died of cerebral edema requiring mechanical ventilation resulting in failure to extubate. Among the patients who survived, 54 (76.1%) had an uneventful recovery, seven (9.8%) developed acute renal failure and required hemodialysis, six (8.5%) developed ARDS, and four (5.6%) developed cerebral edema.

We evaluated the characteristics of the patients who recovered from DKA with those who succumbed to death because of it. Older age group, signs of DMS, pH less than 6.5, high serum potassium, and low serum bicarbonate at the time of presentation, high initial insulin requirement, and high RBS even after the first 12 hours were the predictors of mortality in our sample. The differences were statistically significant as shown in Table 3.

**Table 3:** Predictors of Outcome of Diabetic Ketoacidosis (N=93) CI: Confidence interval, DMS: Depressed mental state, IU: International unit, mg/dL: milligrams per deciliter, L: Liter, meEq/L: milliequivalents per liter, mmol/L: millimoles per liter.

Predictors of outcome	Outcome		Odd’s ratio (CI)	P value
	Recovery (n=71)	Mortality (n=22)		
Age				
Less than 18 years	42 (59.1%)	3 (13.6%)	10.7 (2.91, 39.32)	0.0001
More than 18 years	29 (40.8%)	19 (86.3%)		
Signs of DMS/comatose at presentation				
Yes	11 (15.5%)	14 (63.6%)	0.1 (0.04, 0.31)	<0.00001
No	60 (84.5%)	8 (36.3%)		
Insulin requirement in the first 12 hours				
<50 IU	61 (85.9%)	10 (45.5%)	7.32 (2.5, 21.41)	<0.0001
>50 IU	10 (14.1%)	12 (54.5%)		
Newly developed fever within the first 24 hours				
Yes	8 (11.3%)	9 (40.9%)	0.18 (0.06, 0.56)	0.001
No	63 (88.7%)	13 (59.1%)		
RBS after the first 12 hours				
<300 mg/dL	49 (69.0%)	5 (22.7%)	7.57 (2.48, 23.14)	0.0001
>300 mg/dL	22 (31.0%)	17 (77.2%)		
Fluid requirement in the first 24 hours				
<6 L	39 (54.9%)	11 (50.0%)	1.22 (0.47, 3.18)	0.68
>6 L	32 (45.1%)	11 (50.0%)		
pH at the time of presentation				
6.5–7.2	28 (39.4%)	3 (13.6%)	4.12 (1.12, 15.24)	0.02
<6.5	43 (60.5%)	19 (86.3%)		
Serum potassium at the time of presentation				
>5.5 mEq/L	23 (32.4%)	15 (68.2%)	0.22 (0.08, 0.62)	0.002
<5.5 mEq/L	48 (67.6%)	7 (31.8%)		
Serum bicarbonate at the time of presentation				
>15 mmol/L	53 (74.6%)	9 (40.9%)	4.25 (1.56, 11.61)	0.003
<15 mmol/L	18 (25.4%)	13 (59.1%)		
Urinary Ketones after the first 12 hours				
No urinary ketones	59 (83.1%)	15 (68.2%)	2.29 (0.77, 6.83)	0.12
+2 or more	12 (16.9%)	7 (31.8%)		

## Discussion

This study has elaborated the clinical spectrum of DKA in young adults. The common clinical presentations include severe vomiting, abdominal pain, and DMS. Infections and missed insulin dose were frequently seen as the predisposing factors. In one-fourth of the patients, this episode of DKA was the first presentation of DM. The predictors of mortality include DMS, markedly low pH, and serum bicarbonate, and high serum potassium at the time of presentation, RBS >300 mg/dl and urine positive for ketones even after 12 hours of medical intervention, >50 IU insulin requirement within the first 12 hours, >6L fluid replenished within the first 24 hours, and new onset of fever within the first 24 hours.

This study has elaborately identified the pattern of clinical and biochemical trends in patients with DKA. It will help identify high-risk patients for critical monitoring and immediate medical interventions to prevent mortality. This study has its limitations too. It did not utilize any validated mortality predicting tool. The study was only conducted in one institute which reduces the diversity of the sample. Although patients were divided into two age groups, the younger age group cannot be generalized for all pediatric population since this hospital is an adult general hospital.

In this study, severe vomiting was the most common presenting sign of DKA followed by abdominal pain and DMS. Vomiting has been reported in as many as 54% DKA patients in other studies [3]. Vomiting and abdominal pain have been reported as very common symptoms of DKA in a Kuwaiti study; however, DMS was rather uncommon [8]. In an Indian study, 50% of DKA patients presented with vomiting, 42% with abdominal pain, and 28% with DMS [9]. In another study, 30% of patients presented with DMS, 43% with abdominal pain, and 63% with nausea vomiting [13]. The percentages are comparable to our results. In a Pakistani study, the most common presenting complaint was respiratory distress in 87% patients followed by vomiting in 77% of patients [10]. Severe vomiting and consequent dehydration and hypotension in DKA are related to sudden onset of catabolism and acidemia. Kussmaul breathing, acetone breath, and severity of abdominal pain are related to the severity of acidosis in blood [6]. DMS has been associated with mortality outcome in these patients [5].

The two most frequent factors predisposing to the development of DKA are an underlying infection and insufficient insulin therapy including skipped/missed doses or taking less than the therapeutic dose [6]. In our study, underlying infections were more common than inadequate insulin dose. UTI was the most common infection in our study followed by lower RTI. Other studies have reported comparable results. In a study from Libya, 35% of participants missed their insulin dose and underlying infection was present in 20% DKA patients [3]. Other studies have shown 21%-66% patients missing their insulin dose and 20%-73% patients of DKA having underlying infections [2,9-10,13]. In 26% of patients in this study, DKA was the first presentation of type 1 DM. Literature has frequently shown undiagnosed cases of DM presenting with DKA. In Indian studies, the frequency of first presentation of DKA has been low (10%-11%) [9,13], however in Pakistani studies the frequency has been as high as 57% [10]. In a Libyan study, the frequency reported is 20%. They also showed that patients with first presentation had a significantly longer duration of symptoms before being diagnosed as compared to those who were known cases of DM [3]. In a Kuwaiti study which registered all newly diagnosed cases of type 1 DM from 2011-13, the incidence of DKA at presentation was 33.6% [8]. DKA, with markedly elevated blood glucose levels, has also been identified as the first presentation of type 1 DM in adult patients [14].

Many studies dealing with the clinical spectrum and biochemical trends of DKA have not reported its outcome and mortality rates. Of the studies that have reported it, the probable rate of mortality in DKA ranges from 3.5% to 12% [3,5,9,11,13]. In a study which compared the profiles of septic and non-septic DKA patients, mortality was reported in as many as 57% of septic patients as compared 16% of non-septic patients and an overall mortality rate of 40% [2]. In a mortality report from United States, the in-hospital case-fatality rate of DKA declined during 2000-2014 at an annual average rate of 6.8% (from 1.1% to 0.4% [63.6% decline overall]) [15]. In comparison to the literature, the mortality rate in this study has been higher (23%). The probable explanation of this finding is that this study was conducted only in one center and cannot be generalized. However, since this center is the hub for almost an entire province of Pakistan, there may be delay in patient presentation due to referrals from primary and secondary care institutions. Patient negligence due to high patient influx and limited healthcare staff can also not be neglected.

Various studies have put forward many clinical biochemical signs as predictors of mortality in DKA. High serum lactate and altered sensorium has been associated with high mortality [2]. In another study, males had 7.93-fold better outcomes than females, decrease in mean APACHE II score and serum phosphate at presentation led to 2.86 and 2.71 fold better outcomes, respectively [16]. Similarly, in another study conducted in Nairobi, the mortality rate in DKA was over 29% with altered level of consciousness and systolic hypotension as predictors of unfavorable outcome [17]. Aspiration, onset of renal failure, DMS, and pneumonia at presentation has also been associated with increased mortality risk in an Indian study [5].

This is a lack of consensus on the predictors of mortality in severe DKA. Studies from different parts of the world have quoted different variables as mortality predictors. This highlights the need to develop and validate a globally applicable and acceptable instrument to predict mortality risk in patients presenting with DKA.

## Conclusions

DKA is not an infrequent endocrinological emergency. The clinical presentation is not stark and vague signs such as generalized fatigue, nausea vomiting, abdominal pain, and DMS should raise suspicion in not only diagnosed cases of type 1 DM but also patients with no known comorbids. Underlying infections and inadequate insulin regimen predispose to acute DKA attack. The predictors of mortality include DMS, markedly low pH and serum bicarbonate, and high serum potassium at the time of presentation, RBS >300 mg/dl and urine positive for ketones even after 12 hours of medical intervention, >50 IU insulin requirement within the first 12 hours, >6L fluid replenished within the first 24 hours, and new onset of fever within the first 24 hours. We recommend large scale controlled trials to study the trend of clinical presentations and outcomes of DKA. We also recommend the development of a standard validated instrument to predict mortality in these patients that must be accepted and applied globally.
